# HECT E3 Ubiquitin Ligase Itch Functions as a Novel Negative Regulator of Gli-Similar 3 (Glis3) Transcriptional Activity

**DOI:** 10.1371/journal.pone.0131303

**Published:** 2015-07-06

**Authors:** Gary T. ZeRuth, Jason G. Williams, Yasemin C. Cole, Anton M. Jetten

**Affiliations:** 1 Cell Biology Section, Division of Intramural Research, National Institute of Environmental Health Sciences, National Institutes of Health, Research Triangle Park, North Carolina, United States of America; 2 Mass Spectrometry Group, Division of Intramural Research, National Institute of Environmental Health Sciences, National Institutes of Health, Research Triangle Park, North Carolina, United States of America; University of Minnesota, UNITED STATES

## Abstract

The transcription factor Gli-similar 3 (Glis3) plays a critical role in the generation of pancreatic ß cells and the regulation insulin gene transcription and has been implicated in the development of several pathologies, including type 1 and 2 diabetes and polycystic kidney disease. However, little is known about the proteins and posttranslational modifications that regulate or mediate Glis3 transcriptional activity. In this study, we identify by mass-spectrometry and yeast 2-hybrid analyses several proteins that interact with the N-terminal region of Glis3. These include the WW-domain-containing HECT E3 ubiquitin ligases, Itch, Smurf2, and Nedd4. The interaction between Glis3 and the HECT E3 ubiquitin ligases was verified by co-immunoprecipitation assays and mutation analysis. All three proteins interact through their WW-domains with a *PPxY* motif located in the Glis3 N-terminus. However, only Itch significantly contributed to Glis3 polyubiquitination and reduced Glis3 stability by enhancing its proteasomal degradation. Itch-mediated degradation of Glis3 required the *PPxY* motif-dependent interaction between Glis3 and the WW-domains of Itch as well as the presence of the Glis3 zinc finger domains. Transcription analyses demonstrated that Itch dramatically inhibited Glis3-mediated transactivation and endogenous *Ins2* expression by increasing Glis3 protein turnover. Taken together, our study identifies Itch as a critical negative regulator of Glis3-mediated transcriptional activity. This regulation provides a novel mechanism to modulate Glis3-driven gene expression and suggests that it may play a role in a number of physiological processes controlled by Glis3, such as insulin transcription, as well as in Glis3-associated diseases.

## Introduction

The Glis family of Krüppel-like zinc finger transcription factors, which is comprised of three members designated Glis1-3, contain a zinc finger domain (ZFD) consisting of five Cys_2_-His_2_ zinc finger motifs that exhibit high homology with the ZFDs of members of the Gli and Zic Krüppel-like zinc finger families [1]. The ZDFs of Glis proteins are required for the recognition of specific DNA sequences, referred to as Glis binding sites (GlisBS), located within the regulatory regions of target genes [1–7]. Genetic aberrations within the *GLIS3* locus are associated with a rare syndrome characterized by neonatal diabetes and hypothyroidism and may include polycystic kidney disease, hepatic fibrosis, glaucoma, and mild mental retardation depending on the nature of the mutation [8,9]. A similar phenotype, including neonatal diabetes, polycystic kidney disease, and hypothyroidism, is observed in mice lacking functional Glis3 [3,4,10]. Moreover, a number of genome-wide-association studies (GWAS) have implicated *GLIS3* as a risk locus for the development of type 1 and type 2 diabetes [11–17]. Additional evidence suggests that Glis3 directly regulates insulin transcription in mature beta cells by binding two GlisBS located within the proximal promoter of the preproinsulin gene [3,5,18]. While the ZFD is involved in DNA binding, transcriptional activation of gene expression by Glis3 is mediated through a transactivation domain located within its C-terminus [1,18–20].

To better understand Glis3 function and its roles in disease it is imperative to identify the proteins that regulate or mediate its transcriptional activity. With the exception of a recent report demonstrating that the co-activator, CBP, interacts with Glis3 as part of a transcriptional activation complex that regulates insulin gene transcription [18], little is currently known about the proteins mediating Glis3 transcriptional activity. Posttranslational modifications (PTMs), including phosphorylation, ubiquitination, and acetylation, are also critical in the regulation of the activity and function of many proteins. Ubiquitination of target proteins, which is mediated through a multi-enzyme cascade involving activating (E1), conjugating (E2), and ligating (E3) enzymes, is implicated in the regulation of many physiological processes and in the onset and progression of several pathologies. Ubiquitination has multiple functions that include proteolytic and nonproteolytic roles. The role of ubiquitination in proteolytic degradation by the 26S proteasome is the best studied and provides an important mechanism to regulate protein levels and protein activity, including transcriptional regulation [21]. To obtain greater insights into the mechanisms of action of Glis3, it is important to identify the post-translational modifications that regulate Glis3 activity and function as well as the proteins that catalyze these changes.

In addition to the centrally positioned ZFD and the activation domain at the C-terminus, Glis3 contains a relatively large N-terminus, the function of which is predominately unknown. Previous reports identified a highly conserved region of ~100 amino acids within Glis3 termed the N-terminal conserved region (NCR) that shares extensive homology with a corresponding region of the downstream effectors of hedgehog signaling, Gli1-3 [19]. In order to obtain further insights into the role of the N-terminus of Glis3 in the regulation of its activity and function, we used two different strategies to identify proteins interacting with the Glis3 N-terminus. In the first strategy, the Glis3 N-terminus (aa 1–480) was isolated by affinity co-immunoprecipitation and analyzed by gel-enhanced liquid chromatography mass spectrometry (GeLC-MS). In the second strategy, we performed yeast two-hybrid (Y2H) analysis using the N-terminus of Glis3 as bait to identify interacting protein partners. The two methods identified several WW-domain containing proteins homologous to the E6 AP carboxyl terminus (HECT) E3 ubiquitin ligase, including members of the Nedd4-family of E3 ligases, Nedd4, Smurf1-2, and Itch/AIP4. Follow up studies demonstrated that Itch, Smurf2, and NEDD4 interacted with Glis3 through their WW-domains and that interaction with Itch resulted in increased polyubiquitination of Glis3 and promoted Glis3 proteolytic degradation. Collectively, our study identifies the E3 ubiquitin ligase, Itch, as a critical regulator of Glis3-mediated transcription by controlling the level of Glis3 protein. Our results suggest that Itch functions as a novel modulator of Glis3-mediated transcriptional regulation and as such might modulate Glis3 target genes in cells in which it plays a critical role, such as the regulation of pancreatic beta cell generation and insulin gene expression as well as in Glis3-associated diseases, such as type 1 and 2 diabetes, and polycystic kidney disease.

## Materials and Methods

### Cells and Growth Conditions

Rat insulinoma INS-1832/13 cells, kindly provided by Dr. H. Hohmeier (Duke University), were maintained in RPMI 1640 supplemented with 10% fetal calf serum, 10 mM HEPES, 2 mM glutamine, 1 mM sodium pyruvate, 100 units/ml penicillin, 100 μg/ml streptomycin, and 50 μM β-mercaptoethanol. HEK293T cells were purchased from ATCC and cultured in DMEM containing 10% FBS supplemented with 10% FBS. FreeStyle 293-F cells were obtained from Life Technologies and grown in FreeStyle 293 Expression Medium.

### Gel-enhanced Liquid Chromatography Mass Spectrometry (GeLC-MS)

In-gel digestion and mass spectrometry were performed essentially as described previously [22]. Briefly, immuno-precipitated proteins were separated by SDS-PAGE and the lanes were digested with trypsin (Promega) for 8 hours using a Progest robotic digester (Genomic Solutions). The resulting peptides were extracted by a series of washes with water, acetonitrile, and formic acid. All supernatants were pooled during the collection process and were lyophilized to dryness. The lyophilized samples were resuspended in 40 μL of 0.1% formic acid. NanoLC-ESI-MS/MS analyses were then performed using an Agilent 1100 nanoLC system on-line with an Agilent XCT Ultra ion trap mass spectrometer with the Chip Cube Interface. 20 μl of the peptide mixture were loaded onto an Agilent C18 chip (75 μm x 43 mm) followed by a 15 minute wash of 5% acetonitrile, 0.1% formic acid. Peptides were eluted by applying a linear acetonitrile gradient over 45 minutes. This was followed by a 5 minute acetonitrile gradient and then a 10 minute hold at 95% acetonitrile, 0.1% formic acid.

A peak list was generated from the data obtained from the nanoLC-ESI-MS/MS analysis using the Data Extractor feature of the Spectrum Mill software from Agilent. The Data Extractor settings included limiting the data search to deconvolved ions observed between 400 and 5000 Da and a retention time between 10 minutes and 50 minutes. The resulting extracted data were then searched against the NCBI human and rodent species limited database using the MS/MS Search function in the Spectrum Mill software. Proteins identified with a distinct summed MS/MS search score greater than 17 were tabulated. At this threshold, the false positive rate is approximately 1–3% as determined by searching against a reversed sequence database.

### Yeast-two-hybrid Analysis

Yeast-two-hybrid analysis was performed by Hybrigenics Inc. (Paris, France). The coding sequence for the Glis3 amino terminus (aa 2–500) was PCR-amplified and cloned into pB27 to encode a Glis3(2–500)-LexA fusion protein. The resulting construct was used as bait to screen a murine pancreatic beta cell (βTC-TET) library constructed in plasmid p6. The mated bait and prey strains were spread on medium lacking histidine, leucine, and tryptophan and supplemented with 5.0 mM 3-aminotriazole to prevent bait auto-activation. 179 clones were processed out of the 130 million analyzed interactions. Prey fragments were amplified at their 5’ and 3’ ends and sequenced. The sequences obtained were used to identify the interacting proteins in the GenBank database (NCBI).

### Generation of Reporter and Expression Constructs

The generation of pCMV10-3xFLAG-Glis3, human GLIS3, truncated mutants of mouse Glis3, pCMV-HA-Ub, and p-mIP-696-Luc was described previously [1,18,19]. The N-terminal region of Glis3 (aa1-480) with an N-terminal FLAG and C-terminal HA tag was generated by subcloning the coding region for FLAG-Glis3 from pCMV10-3xFLAG-Glis3 into pCMV6-HA (Origene) using the *Nhe*I and *Mlu*I restriction sites. Next, the coding region for FLAG-Glis3-HA was PCR amplified and subcloned into pIRES2-EGFP (Clontech) using the *Sal*I and *Bam*HI restriction sites. pCMV10-3xFLAG-Glis3 was created by PCR amplification of the *Danio rerio* glis3 coding region followed by subcloning it into pCMV10-3xFLAG (Sigma) using the *Eco*RI and *Bam*HI restriction sites. pCMV10-3xFLAG-Glis3-ΔC480 was created by PCR amplifying the region of Glis3 encoding amino acid 1–480 and subcloning it into pCMV10-3xFLAG using the *Eco*RI and *Bam*HI restriction sites. pRK-Myc Smurf2 was generated by Ying Zhang [23] and purchased from Addgene (plasmid 13678). To generate p-CMV-Myc-NEDD4 and p-CMV-Myc-Itch the respective coding regions were amplified by PCR and subcloned into pCMV-Myc (Clontech) using the *Bgl*II and *Kpn*I restriction sites. The Myc-tagged WW-domains of Itch (aa 274–477), Smurf2 (aa 143–334), and NEDD4 (aa 238–497) and Itch-Δ-C2 (aa 274–806) and Itch-Δ-HECT (aa 1–477) were made by amplifying the indicated regions by PCR and their subsequent subcloning into pCMV-Myc using the *Bgl*II and *Kpn*I restriction sites. FLAG-Glis3-PY^461^ mut, FLAG Glis3-ZF muts, pCMV-HA-Ub muts, pCMV-Myc-Smurf2-C716G, Itch-C832G, and NEDD4-C854G were generated by *in vitro* site-directed mutagenesis as described previously [18] using the primers listed in S1 Table.

The HA-tagged ubiquitin KO with all lysines mutated to arginine was generated by Ted Dawson [24] and purchased from Addgene (plasmid #17603). pCMV10-3xFLAG-3xNLS-Glis3-ΔC480 was generated by inserting a double stranded 3xNLS sequence (5’*AGCTT*GATCCAAAAAAGAAGAGAAAGGTAGATCCAAAAAAGAAGAGAAAGGTAGATCCAAAAAAGAAGAGAAAGGTA*GC* with *HindIII* and *NotI* overhangs in-frame and upstream of the Glis3 ΔC480 coding sequence in pCMV10-3xFLAG. pCMV10-3xFLAG-G4DBD-Glis3-ΔC480 was made by PCR amplifying the Gal4(DBD) (aa 1–147) and inserting it in-frame upstream of the Glis3 ΔC480 coding sequence in pCMV10-3xFLAG using *Not*I and *Eco*RI restriction sites.

The HEK293-F stable cell lines were made by transfecting adherent cells with either pIRES-EGFP2-FLAG-Glis3-HA or empty vector and treating cells with 1 mg/ml Geneticin (Life Technologies) for 2 weeks. Surviving colonies were pooled and maintained in 250 μg/ml Geneticin. Expression of EGFP was measured by fluorescence microscopy and expression of a single protein product of the correct weight was determined by Western blotting using anti-M2 FLAG-HRP (Sigma) and anti-HA (Roche) antibody.

### Transfection and reporter assays

Cells were plated in 12-well dishes at 1 × 10^5^ cells/well and 24 h later transfected in Opti-MEM (Invitrogen) with pCMV-β-galactosidase and the indicated plasmids using either Lipofectamine 2000 (Invitrogen) for HEK293T cells or Lipofectamine LTX with PLUS reagent for INS1 cells following the manufacturer’s protocol. After 24 h, cells were harvested and luciferase and β-galactosidase activities measured using a luciferase assay kit (Promega) and a luminometric β-galactosidase detection kit (Clontech), respectively, following the manufacturer's protocols. Each data point was assayed in triplicate and each experiment was performed at least twice. Relative luciferase activity was calculated. All values underwent analysis of variance and Tukey-Kramer comparison tests using InStat software (GraphPad Software Inc.), and data are presented as mean ± S.E.

### Co-Immunoprecipitation Assays

Cells were harvested by scraping in radioimmunoprecipitation assay buffer (25 mM Tris, 150 mM NaCl, 1 mM EDTA, 1 mM EGTA, 20 mm sodium molybdate, and 0.5% Nonidet P-40) containing protease inhibitor cocktails I and II (Sigma). Cell lysates were centrifuged at 16,000 × *g* for 10 min at 4°C. A portion of the supernatants was incubated sequentially at RT for 10 min with Dynabeads (Invitrogen) conjugated to anti-Myc antibody (Invitrogen), high affinity anti-HA antibody (Roche) or anti-M2 FLAG antibody (Sigma). Magnetic beads were washed three times with 200 μl of ice-cold PBS (137 mM NaCl, 10 mM phosphate, 2.7 mM KCl, pH 7.4). Bound protein complexes and input fractions were examined by Western blot analysis using mouse-anti-Myc (Invitrogen), mouse anti-FLAG (Sigma), rat anti-HA (Roche), or mouse anti-ITCH (BD Transduction Laboratories) antibodies.

### In vitro pulldown assay

The coding regions for FLAG-Glis3 or the *PY* ^*461*^ mutant and Myc or Myc-Itch were subcloned into TOPO2.1 (Invitrogen) following the manufacturer’s protocol. The resulting plasmids were used to synthesize protein *in vitro* using the TNT Quick Coupled Transcription Translation kit (Promega). 5 μl of Myc or Myc-Itch was added to 415 μl PBS supplemented with protease inhibitor cocktail and incubated with Dynabeads (Life Technologies) conjugated to anti-Myc antibody (Invitrogen) for 30 min at RT. The beads were washed 3x with PBS and 5 μl FLAG Glis3 or *PY* ^*461*^ mut was added to 415 μl PBS supplemented with protease inhibitor and incubated with the beads overnight at 4°C. The beads were then washed 3x with PBS and proteins were eluted in 1x Laemmli buffer containing β-mercaptoethanol by boiling for 5 minutes. Proteins were separated by SDS-PAGE and analyzed by Western blotting using anti-M2 FLAG-HRP antibody.

### Cell Fractionation

Cells were plated on 150 mm dishes and transfected as described above. After 48 h, cells were washed 3x with ice cold PBS (137 mM NaCl, 2.7 mM KCl, 10 mM Na_2_HPO_4_, 1.2 mM KH_2_PO_4_) and resuspended in hypotonic buffer (10 mM Tris pH 7.9, 1.5 mM MgCl_2_, 10 mM KCl, 0.5 mM DTT, 0.2 mM EDTA) supplemented with protease inhibitor cocktail (Sigma) for 15 minutes. Plasma membranes were lysed by the addition of Nonidet P-40. Cytoplasmic proteins were collected in the supernatant after nuclei were pelleted by centrifugation. Nuclei were washed in hypotonic buffer and resuspended in nuclear extraction buffer (25% glycerol, 20 mM Tris pH 7.9, 1.5 mM MgCl_2_, 800 mM (NH_4_)_2_SO_4_, 0.2 mM EDTA) supplemented with protease inhibitor cocktail for 30 min at 4°C. Nuclear proteins were collected in the supernatant after pelleting debris by centrifugation.

### Fluorescence microscopy

Cells were transfected with the indicated plasmids as described above. After 24 h, cells were washed 5x with ice cold PBS and fixed in 4% paraformaldehyde in PBS for 20 min at RT. Cells were permeabilized with Triton-x 100 for 7 min and subsequently blocked with Superblock (Pierce) for 15 min at RT. Cells were stained with primary antibody for 3 h and secondary stained with anti-mouse or anti-rat Alexa-488 for 30 min. Cells were washed with PBS containing 0.1 μg/ml DAPI. Imaging was performed using an Olympus IX-70 inverted fluorescence microscope.

### Quantitative Reverse Transcriptase Real-time PCR Analysis

RNA was isolated from INS-1(832/13) cells 48 h after transfection using an RNeasy mini kit (Qiagen) according to the manufacturer’s specifications. Equal amounts of RNA were used to generate cDNA using a high capacity cDNA kit (Applied Biosystems), and cDNA was analyzed by quantitative real-time PCR. All qRT-PCR was performed in triplicate using a StepOnePlus real-time PCR system (Applied Biosystems). For r*Ins2*, Power SYBR Green PCR Master Mix (Applied Biosystems) was used with 25 ng of cDNA per reaction, forward primer, 5**’**-CAGCAAGCAGGAAGCCTATC, and reverse primer, 5’-TTGTGCCACTTGTGGGTCCT. The average Ct from triplicate samples was normalized against the average Ct of 18S rRNA.

### Western Blot Analysis and Protein Quantification

Proteins were resolved by SDS-PAGE and then transferred to PVDF membrane (Invitrogen) by electrophoresis. Immunostaining was performed with the indicated antibody at either 4°C for 18 h or 22°C for 2 h in BLOTTO reagent (5% nonfat dry milk dissolved in 50 mM Tris, 0.2% Tween 20, and 150 mM NaCl). Blots were subjected to three 15-min washes in TTBS (50 mM Tris, 0.2% Tween 20, and 150 mM NaCl), and proteins were detected by enhanced chemiluminescence following the manufacturer’s protocol (GE Healthcare). Proteins were quantified using ImageQuant TL software analysis (GE Healthcare). The mean intensity of the experimental bands minus the background were normalized against the mean intensity of GAPDH bands minus the background. All samples were run in triplicate and all experiments performed at least three times independently. Data are presented as mean ± S.E.

## Results

### Identification of Glis3 interacting partners by GeLC-MS and Y2H analysis

**Fig 1:**
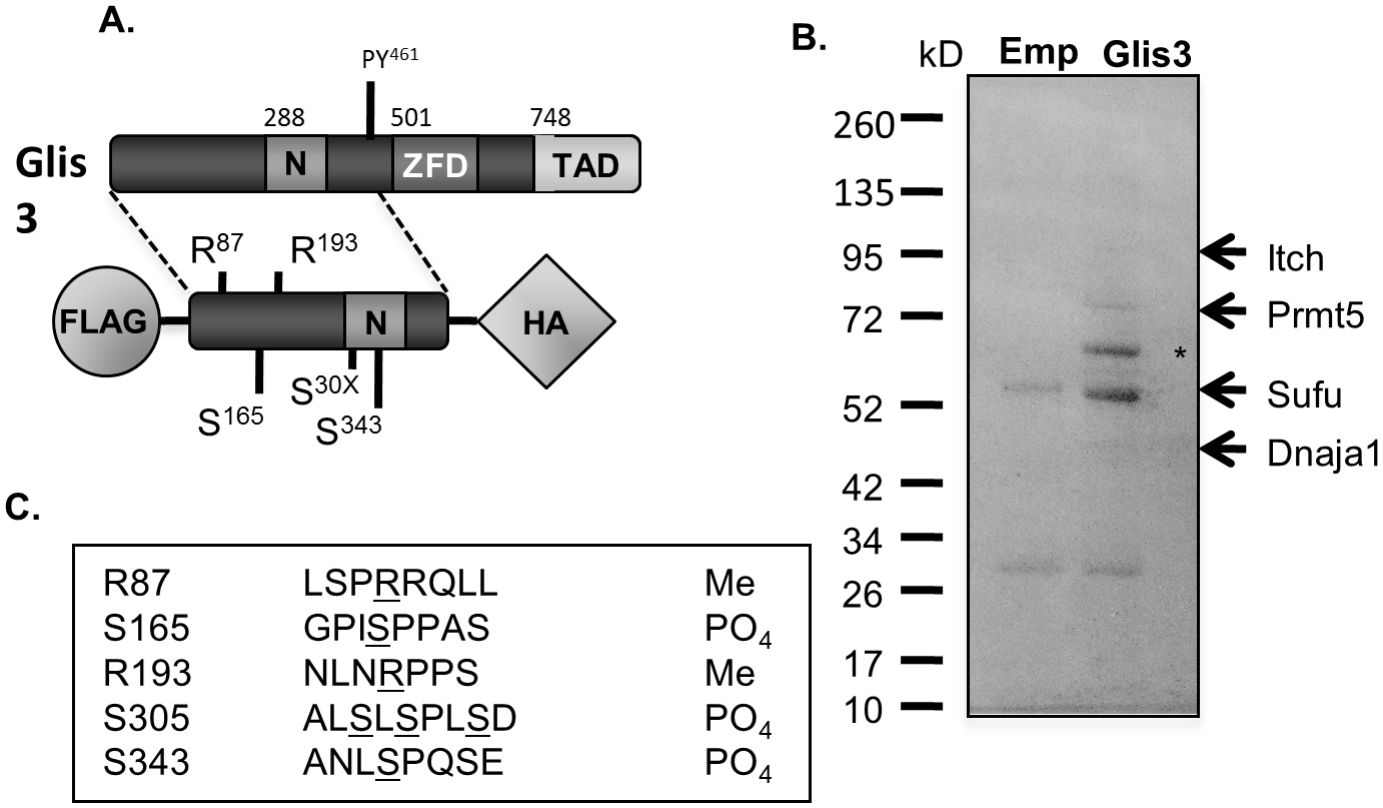
Mass spectrometry analysis identified several putative Glis3-interacting proteins and Glis3 post-translational modifications. A. Schematic representation of dual tagged Glis3 N-terminus stably expressed in HEK293F cells used for the tandem affinity pulldown and GeLC-MS analysis. Posttranslationally modified sites identified by MS analysis are indicated. N = N-terminal conserved region; ZFD = zinc finger domain; TAD = transactivation domain. B. Representative Coomassie blue stained gel showing proteins immunoprecipitated by a tandem FLAG/HA pulldown. Protein complexes from HEK293F cells stably expressing pIRES2-EGFP-FLAG-HA empty vector or pIRES2-EGFP-FLAG-Glis3-ΔC480-HA were immobilized using anti-M2 FLAG and anti-HA antibody conjugated to agarose or magnetic beads, respectively. Eluted proteins were separated by SDS-PAGE and the gel was stained with Coomassie blue and analysed by GeLC-MS. Arrows indicate bands corresponding to approximate molecular weight of Glis3 interacting proteins identified by MS. * indicates over-expressed FLAG-Glis3-ΔC480-HA. C. Table showing posttranslational modifications of Glis3-ΔC480 identified by MS analysis. Modified amino acid positions are underlined. Specificity could not be determined between phosphorylation at S305, 307, or 310. Me = methylation; PO_4_ = phosphorylation.

To determine the potential importance of the 500 aa N-terminal region of Glis3 in regulating the function of the protein, tandem affinity purification (TAP) followed by gel-enhanced liquid chromatography mass spectrometry was used to identify partners that may interact with the region. The N-terminal region up to amino acid 480, including an N-terminal 3xFLAG and a C-terminal HA epitope (Fig 1A), was stably expressed in HEK293-F cells. Subsequently, TAP was performed on cell lysates and eluted proteins were separated by SDS-PAGE and analyzed by Coomassie blue staining. In addition to Glis3 (indicated by an * in Fig 1B), GeLC-MS identified a number of proteins that potentially interact with the Glis3 N-terminus, including SUFU, a previously verified interacting partner [19]. Other putative partners of interest that were identified with high confidence in more than one experiment included protein arginine methyltransferase 5 (Prmt5) and Itchy E3 ubiquitin protein ligase (Itch). S2 Table contains a full list of co-immunoprecipitated proteins. Further, GeLC-MS identified several putative post-translational modifications (PTM), two phosphorylation and two methylation sites, in the Glis3 N-terminus (Fig 1C).

As an alternative strategy to identify Glis3 interacting proteins, we performed a yeast 2-hybrid (Y2H) screen using the Glis3 N-terminus (aa 1–480) as bait. Since Glis3 is expressed in pancreatic beta cells and is important for mature beta cell function, including insulin production [3–5, 18, 25], a murine pancreatic beta cell library was used as prey. Selected high confidence interacting partners that were identified are listed in Table 1 along with the domain involved in mediating the interaction with the Glis3 N-terminus. Itch was identified by both GeLC-MS and Y2H analysis as a potential Glis3 interacting protein. Interestingly, in addition to Itch, several other closely related members of the WW-domain containing HECT E3 ubiquitin ligase family, including neural precursor cell expressed developmentally down-regulated protein 4 (Nedd4) and SMAD ubiquitination regulatory factor 2 (Smurf2), were identified as potential interacting partners through their WW domains. The identification of different interacting partners by the two methods might relate to the different cell types used in TAP and Y2H analysis and the fact that in Y2H analysis protein fragments are used as prey to identify Glis3 interacting partners, whereas in TAP full-length endogenous proteins are pulled down and analyzed. In addition, differences in subcellular localization and posttranslational modifications may be contributory factors.

**Table 1:** Selected list of putative interacting Glis3 partners identified by yeast 2-hybrid analysis. Protein name is followed by the amino acid region of the protein found to interact with the Glis3 N-terminus as well as the protein domain corresponding to that region. The right column shows the number of distinct clones that interacted with Glis3 as well as the total number of interactions.

Protein	Region	Interacting Domain	# different clones
Dnaja1	(205–433)	HSP40/DnaJ peptide-binding	2 (2 hits)
Hipk3	(156–541)	Serine/threonine protein kinase	6 (7 hits)
Isl1	(106–347)	Homeobox	2 (3 hits)
Itch	(224–462)	WW/Rsp5/WWP	2 (5 hits)
Kdm4c	(721–800)	Zinc finger, PHD-type	2 (4 hits)
Nedd4	(393–444)	WW/Rsp5/WWP	9 (17 hits)
Smurf1	(199–383)	WW/Rsp5/WWP	1 (6 hits)
Smurf2	(150–328)	WW/Rsp5/WWP	1 (2 hits)
Spop	(33–237)	MATH/TRAF-like	3 (3 hits)
Wwp2	(395–453)	WW/Rsp5/WWP	1 (2 hits)
Zmym2	(366–696)	Zinc finger, MYM-type	3 (3 hits)

### Glis3 interacts with HECT E3 ubiquitin ligases

**Fig 2:**
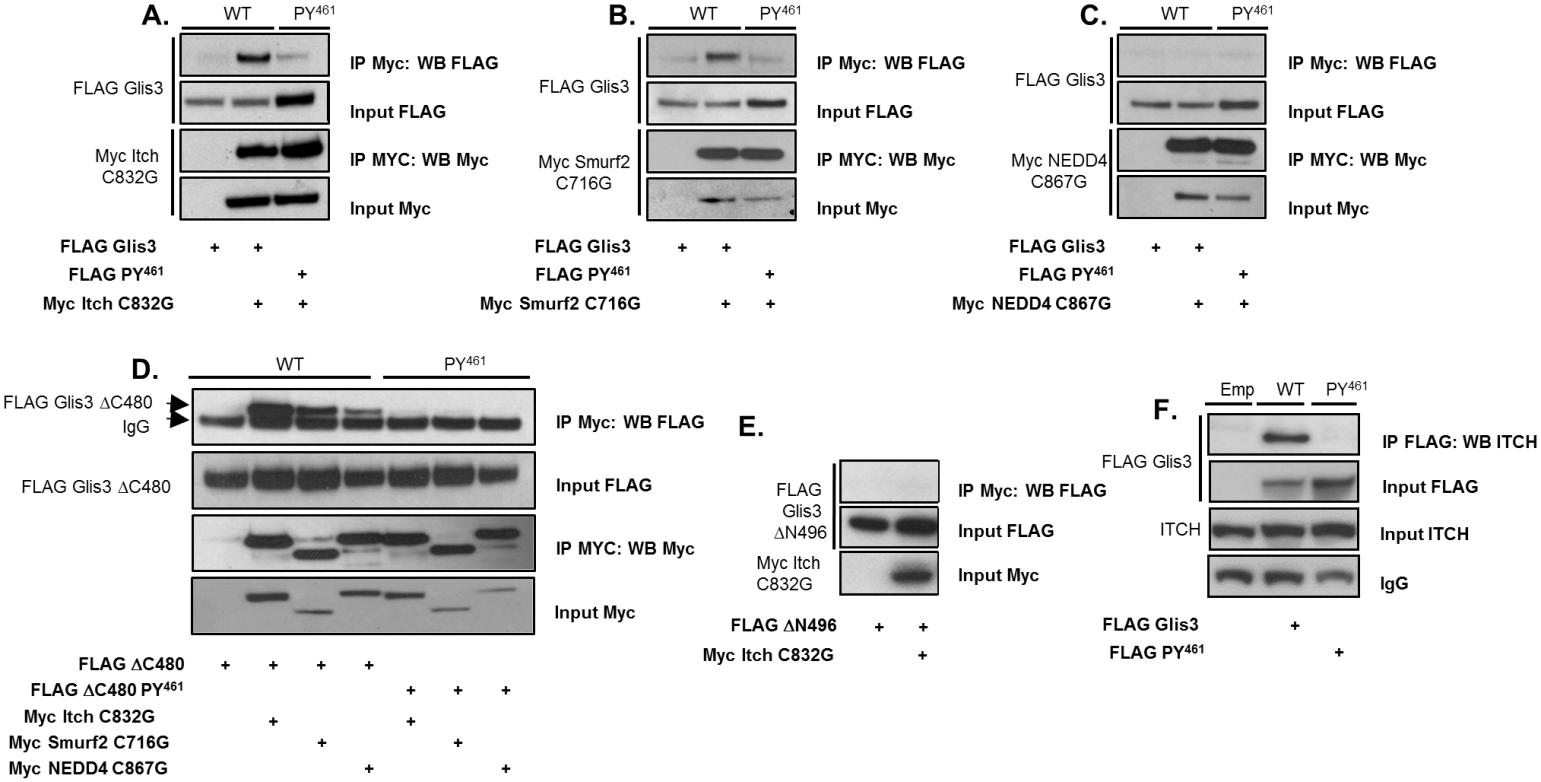
Glis3 associates with Itch, Smurf2, and NEDD4. A-C. HEK293T cells were transfected with FLAG-Glis3 or the FLAG-Glis3-*PY* ^*461*^ mutant and Myc empty vector, Myc-Itch-C832G, Myc-Smurf2-C716G, or Myc-NEDD4-C867G as indicated. Co-immunoprecipitation was performed using a mouse anti-Myc antibody and immunoprecipitated proteins were examined by Western blot analysis using anti-M2 FLAG-HRP or anti-Myc and goat anti-mouse-HRP antibodies. D. HEK293T cells were transfected with FLAG-Glis3-ΔC480 or its respective *PY* ^*461*^ mutant and Myc empty vector, Myc-Itch-C832G, Myc Smurf2-C716G, or Myc-NEDD4-C867G and co-IPs performed as described for A-C. E. HEK293T cells were transfected with FLAG-Glis3-ΔN496 and Myc empty vector or Myc-Itch-C832G and co-IPs performed as described in A-C. F. HEK293T cells were transfected with FLAG empty vector, FLAG-Glis3 or the FLAG-Glis3-*PY* ^*461*^ mutant as indicated. After 48 h co-immunoprecipitation was performed using a mouse anti-M2 FLAG antibody and immunoprecipitated proteins were examined by Western blot analysis using mouse anti-ITCH primary and goat anti-mouse-HRP antibodies.

Since several HECT E3 ubiquitin ligases were identified several times as possible Glis3 interacting proteins, we focused our study on the further characterization of these interactions. Co-immunoprecipitation was performed using HEK293T cells co-expressing FLAG-tagged Glis3 (p-CMV-3xFLAG-Glis3) and Myc-tagged Itch, Smurf2, or NEDD4, the putative WW-domain interacting proteins identified with the highest level of confidence in the Y2H analysis. Since co-expression of Itch with Glis3 resulted in substantially decreased levels of Glis3 protein, a catalytically inactive Itch mutant (C832G) was used to study the interaction [26]. Western blot analysis showed that Itch and Smurf2 co-immunoprecipitated with Glis3 (Fig 2A and 2B) although the interaction appeared much weaker with Smurf2 than with Itch. NEDD4 failed to detectably interact with Glis3 by co-IP (Fig 2C). WW-domains are known to interact with proteins through the recognition of proline-rich motifs, including PPLP or PPxY motifs or proline residues preceded by phosphorylated serine or threonine (pSP or pTP motifs) [27–30]. Examination of the N-terminal sequence of Glis3 revealed a single PPxY motif located between aa 458–461 (PY^461^) as well as 18 putative p(S/T)P motifs. The Glis3 PY^461^ motif was conserved across all species examined ranging from fishes to humans (S1A Fig). Mutation of the PY^461^ motif dramatically reduced Glis3 interaction with Itch and Smurf2 (Fig 2A and 2B).

To determine whether the three ubiquitin ligases were capable of interacting with the N-terminus of Glis3 alone, as was used in the GeLC-MS and Y2H analyses, the co-IP was repeated in HEK293T cells expressing FLAG-Glis3-ΔC480 and each of the Myc-tagged WW-domain-containing proteins. As seen in Fig 2D, all three of the proteins were capable of interacting with the N-terminus of Glis3 consistent with the results of MS and Y2H. As observed using the full-length Glis3, the interaction was strongest with Itch and decreased with Smurf2 and NEDD4, respectively. Mutation of the PY^461^ motif totally abrogated the interaction with Itch, Murf2, and NEDD4 (Fig 2D).

Glis3 contains one additional PPxY motif located between aa 838–841 (PY^841^) located in the C-terminus of the protein. Study of the effect of mutation of the PY^841^ motif either alone or in combination with the PY^461^ mutation on the interaction of Glis3 with Itch indicated that it did not play any role in mediating this interaction (S1B Fig). Consistent with this is the finding that Itch was not capable of interacting with FLAG-Glis3-ΔN496, containing only the C-terminal half of Glis3 (Fig 2E). These data suggest that the PY^461^ motif is responsible for meditating the interaction between Glis3 and the WW-domain containing HECT E3 ubiquitin ligases, but the affinities of the interactions likely vary between the three proteins. Since both Glis3 and the HECT E3 ligases are being overexpressed at supraphysiological levels, it is likely that the high concentrations of the proteins may force interactions that would not occur at physiological levels of expression. To diminish that concern, we examined the interaction of exogenously expressed FLAG-Glis3 or its PY^461^ mutant in HEK293T cells with endogenous ITCH protein using immunoprecipitation and an anti-Flag M2 antibody. Indeed, as shown in Fig 2F, endogenous ITCH interacted robustly with Glis3, while no interaction was detected with the PY^461^ mutant, reinforcing the requirement for that motif. Although several commercial Glis3 antibodies are available none of these were able to recognize endogenous Glis3 protein thereby preventing us from examining the interaction with endogenous Glis3.

### Interaction between Glis3 and HECT E3 ubiquitin ligases is mediated by WW-domains

**Fig 3:**
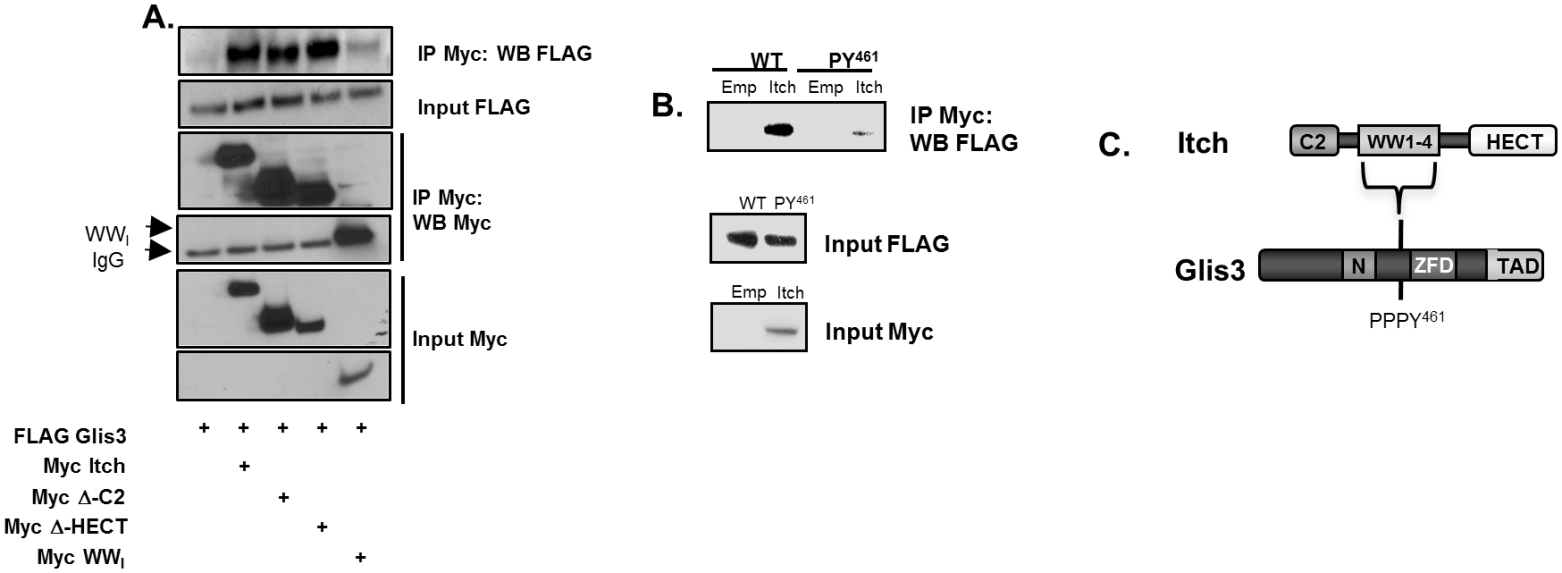
Itch directly interacts with Glis3 through its WW-domains. A. HEK293T cells were transfected with FLAG-Glis3 and Myc empty vector, Itch, Itch-Δ-C2, Itch-Δ-HECT, or Itch-WW_I_ containing only the four WW-domains. Co-immunoprecipitation was performed as described in Fig 2A–2C. B. FLAG-Glis3 or the *PY* ^*461*^ mutant and Myc Empty or Myc-Itch were transcribed and translated *in vitro* and an *in vitro* pulldown assay was performed using anti-Myc antibody as described in Methods and Materials. G. Schematic showing the interaction between Itch with the *PY* ^*461*^ motif of Glis3.

Members of the Nedd4/Rsp5p family contain in addition to the centrally located WW-domains, an N-terminal C2 domain and a C-terminal HECT domain [31]. To determine whether these domains influence the interaction between Itch and Glis3, IP was performed with Glis3 and Itch lacking its C2 or HECT domains. The results showed that deletion of either the C2 or the HECT domain (Fig 3A) had little effect on the interaction between Itch and Glis3 and therefore are not required for the interaction. In addition, the four WW domains from Itch were sufficient for interaction with Glis3 although the interaction was considerably weaker than observed with full-length Itch and might be due to differences in the WW domain conformation and therefore affinity for Glis3 between full-length Itch and the Itch WW domain only. The WW-domains of Smurf2 and NEDD4 were also sufficient for interaction with Glis3 in a *PY* ^*461*^ motif dependent manner (S1C and S1D Fig). These data indicate that the association of Glis3 with these HECT ubiquitin ligases is mediated through a direct interaction of the WW domains with the *PY* ^*461*^-motif of Glis3. In order to determine whether the interaction required secondary proteins associated with either Itch or Glis3, an *in vitro* pulldown was performed using *in vitro* transcribed and translated proteins (TnT-FLAG Glis3 and TnT-Myc Itch). As seen in Fig 3B, TnT-Myc-Itch immunoprecipitated with TnT-FLAG Glis3 and a weaker interaction was observed between TnT-Myc Itch and TnT-FLAG *PY* ^*461*^ mutant suggesting that the interaction is direct and does not require intermediate proteins. Collectively, these results demonstrate that the HECT E3 ubiquitin ligases interact directly with the *PY* ^*461*^ motif of Glis3 through their WW-domains and that mutation of two residues of the core PPxY motif greatly, but not completely, disrupt their interaction. A schematic summary of the interaction between Glis3 and Itch is shown in Fig 3C.

### HECT E3 ubiquitin ligases promote Glis3 polyubiquitination

**Fig 4:**
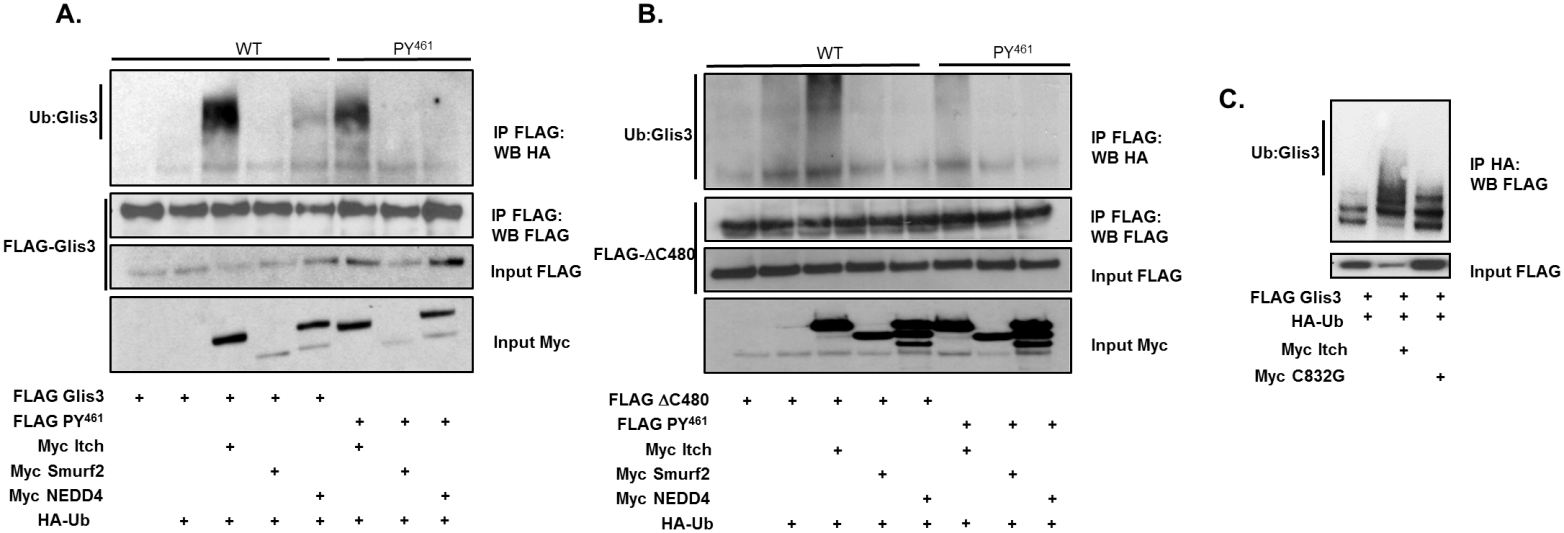
Itch, Smurf2, and NEDD4 polyubiquitinate Glis3. A-B. HEK293T cells were transfected with CMV-HA-Ubiquitin, FLAG-Glis3 or FLAG-Glis3-ΔC480 or their respective *PY* ^*461*^ mutants, and Myc-Itch, Smurf2, NEDD4, or empty vector as indicated. Cells were treated with 10 μM MG132 for 6 h prior to harvest. Co-immunoprecipitation was performed using an anti-M2 FLAG antibody and immunoprecipitated proteins were analysed by Western blot using a high affinity anti-HA, anti-M2 FLAG-HRP, anti-Myc or goat anti-mouse-HRP antibodies. C. HEK293T cells were transfected with FLAG Glis3, CMV-HA-Ubiquitin, and Myc-Itch or its catalytically inactive mutant as indicated. Co-IP was performed as described in A-B. D. HEK293T cells were transfected with FLAG-Glis3, Myc Itch, and HA-Ubiquitin or the K48R or K63R ubiquitin mutants as indicated. Co-IP was performed as described in A-B.

To determine whether the E3 ubiquitin ligases were capable of promoting Glis3 ubiquitination as a result of their interaction, FLAG-Glis3 or the *PY* ^*461*^ mutant was co-expressed in HEK293T cells with HA-tagged ubiquitin and Myc-tagged Itch, Smurf2, NEDD4, or empty vector and the effect on Glis3 ubiquitination examined. As seen in Fig 4A, only Itch substantially enhanced Glis3 ubiquitination. Furthermore, consistent with the data in Fig 1, mutation of the *PY* ^*461*^ motif significantly reduced, but did not totally eliminate Itch-mediated ubiquitination of Glis3 (Fig 4A and S2A Fig). The latter is consistent with our observation that mutation of the *PY* ^*461*^ motif did not totally abolish the interaction with Glis3 (Fig 2B) suggesting that Itch might weakly interact with Glis3 through an additional flanking residues. Likewise, Itch also enhanced the ubiquitination of Glis3-ΔC480, which was greatly reduced by mutation of the *PY* ^*461*^ motif (Fig 4B and S2B Fig). Again, Smurf2 and NEDD4 had little effect on the ubiquitination of Glis3-ΔC480. To establish whether the effect of Itch on Glis3 ubiquitination was due to its ability to transfer ubiquitin, the catalytic cysteine in the HECT domain was mutated and its ability to promote Glis3 ubiquitination was assessed. Fig 4C shows that the catalytically inactive mutant was virtually incapable of promoting Glis3 ubiquitination. Finally, in order to determine what type of polyubiquitin chain is transferred to Glis3 by Itch, HA-ubiquitin containing a mutation that converts either Lys^48^ or Lys^63^ to Arg was used in a pulldown with Itch and Glis3. The results indicate that mutation of ubiquitin K48 had only a small effect on Glis3 polyubiquitination by Itch, while the K63R ubiquitin mutation significantly reduced Itch-mediated Glis3 ubiquitination, but did not totally eliminate it (S3 Fig). Collectively these data indicate that Itch induces K63-linked rather than K48-linked polyubiquitination of Glis3 and that this process is dependent upon the catalytic domain of Itch.

### Itch promotes proteolytic degradation of Glis3

**Fig 5:**
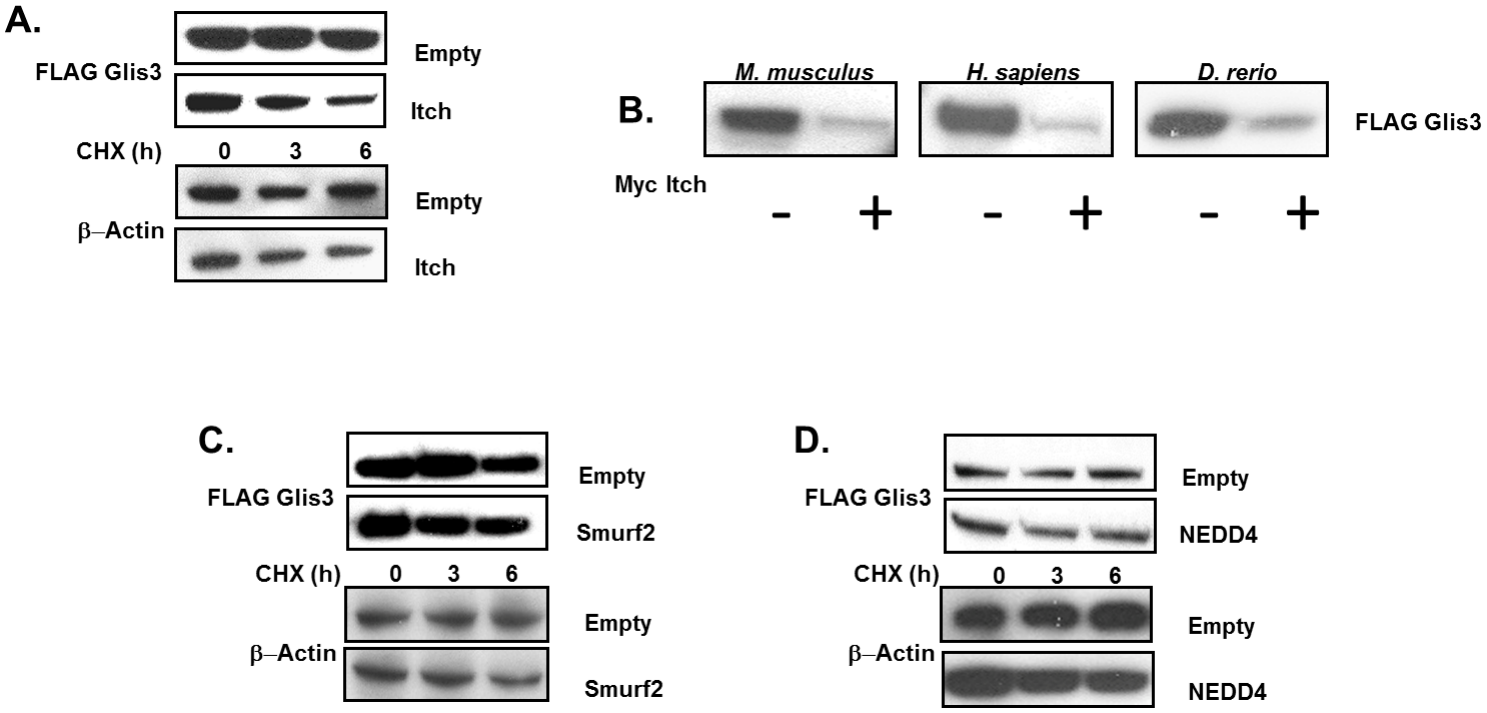
Itch targets Glis3 for degradation. A. HEK293T cells were transfected with FLAG-Glis3 and Myc-Itch, or empty vector as indicated. Prior to harvest, cells were treated with 10 μg/ml cycloheximide for the indicated duration. Proteins were analysed by Western blotting using anti-M2 FLAG-HRP antibody, anti-Myc and goat anti-mouse-HRP antibodies, or anti-β actin and goat anti-rabbit-HRP antibodies. Bands were quantified as described in Materials and Methods. B. HEK293T cells were transfected with mouse, human, or zebrafish Glis3 and co-transfected with Myc-Empty vector or Myc-Itch as indicated. Glis3 protein levels were assessed by Western blot using anti-M2 FLAG-HRP antibody. C-D. HEK293T cells were transfected with FLAG-Glis3 and Myc-Smurf2, -NEDD4, or empty vector as indicated. Cells were treated and harvested and proteins analysed as described in A.

**Fig 6:**
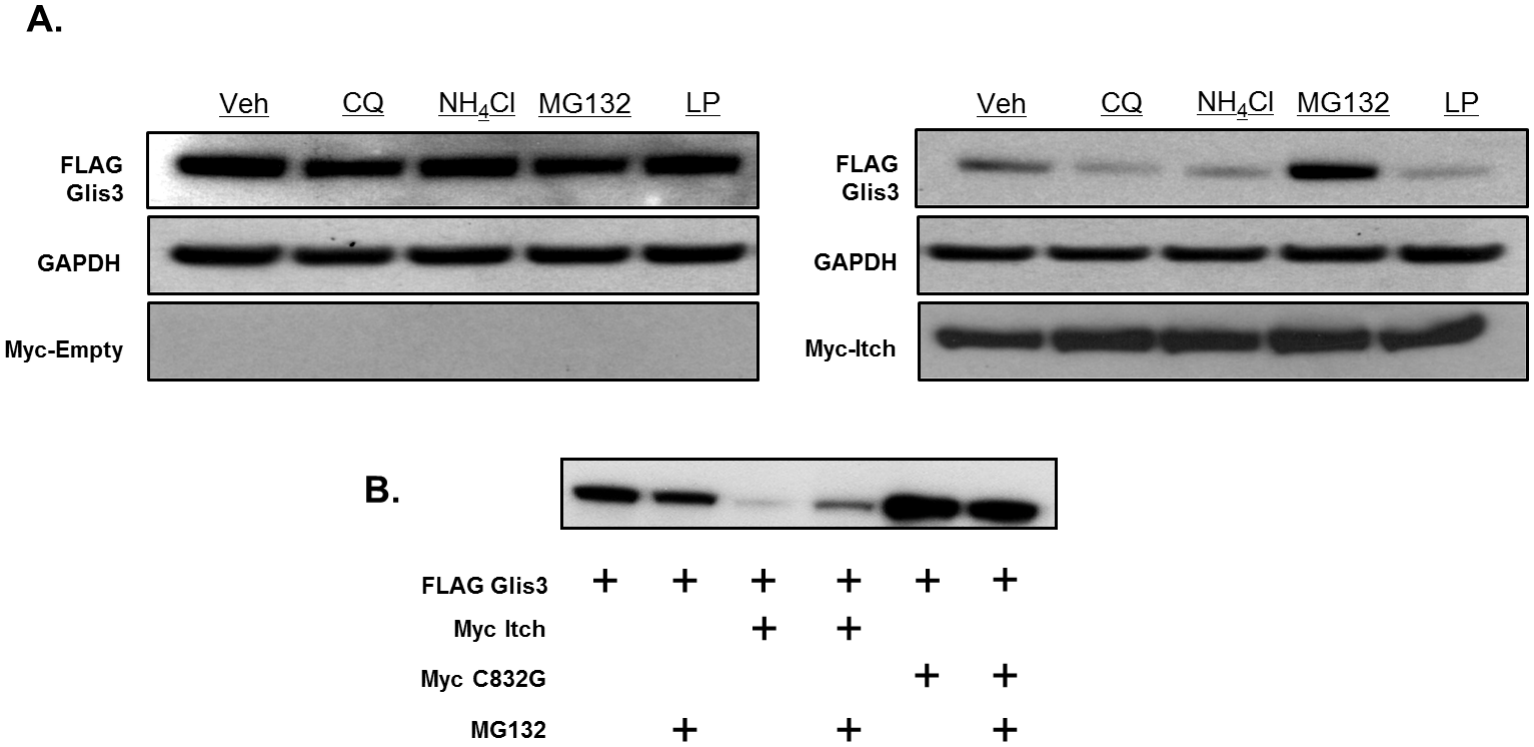
Itch targets Glis3 for degradation through the 26s proteasome. A. HEK293T cells were transfected with FLAG-Glis3 and Myc-Itch or empty vector. Cells were treated with DMSO, 10 μM MG132, 10 mM ammonium chloride, 200 μM chloroquine, or 100 μM leupeptin as indicated for 7 h. Proteins were analysed by Western blotting using anti-M2 FLAG-HRP antibody or anti-Myc and goat anti-mouse-HRP antibodies. GAPDH is shown as a loading control. B. HEK293T cells were transfected with FLAG-Glis3 and Myc-Itch, catalytically inactive Myc-Itch C832G, or empty vector. Cells were treated with 10 μM MG132 or DMSO for 6 h as indicated. Proteins were analysed by western blotting using an anti-M2 FLAG-HRP antibody.

Given that Itch interacted with and promoted Glis3 ubiquitination, we examined whether this targeted Glis3 for proteolytic degradation. We co-transfected HEK293T cells with FLAG-Glis3 and Myc-tagged-Itch, -Smurf2, -NEDD4, or empty vector and monitored Glis3 protein stability by Western blot analysis after timed cycloheximide treatment. Co-expression of Itch significantly reduced murine Glis3 protein stability relative to cells transfected with empty vector and internal controls (Fig 5A). Murine Itch was likewise capable of promoting the degradation of human GLIS3 as well as *Danio rerio* glis3 suggesting that the function is likely conserved throughout vertebrate evolution (Fig 5B). As expected, Smurf2 and NEDD4 did not have any significant effect on Glis3 protein stability (Fig 5C and 5D). Protein ubiquitination typically promotes degradation by targeting the ubiquitinated proteins to the 26S proteasome or less frequently, the lysosome [32]. To determine by which mechanism Itch promoted Glis3 degradation, the effect of lysosomal or proteasomal inhibitors on Glis3 protein degradation was examined. As seen in Fig 6A, the general proteasome inhibitor MG132 significantly reduced degradation of Glis3 by Itch, whereas the lysosomal inhibitors, NH_4_Cl and chloroquine, failed to protect Glis3 against Itch-directed degradation as did leupeptin, an inhibitor of trypsin-like and cysteine proteases (Fig 6A). Furthermore, while MG132 stabilized Glis3 protein expression in the presence of exogenous Itch, it had no observable effect on Glis3 co-expressed with the catalytically inactive Itch mutant (Fig 6B). Collectively, these results suggest that Itch targets Glis3 for degradation *via* the 26S proteasome.

### The Glis3 ZFD is required in addition to the PY^461^ motif for Itch-mediated degradation

**Fig 7:**
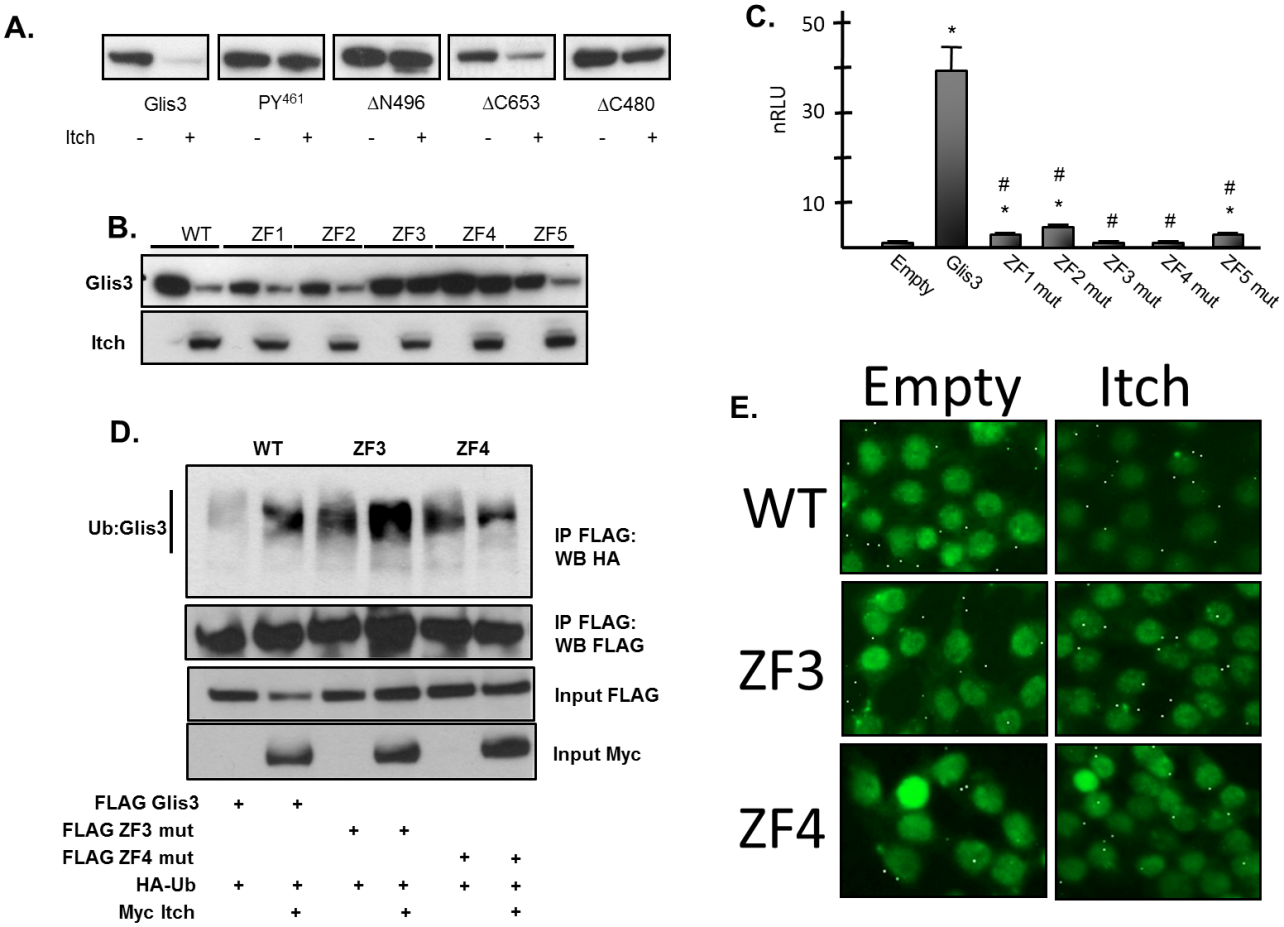
The *PY* ^*461*^ motif and the ZFD are required for Itch-mediated degradation of Glis3. A. HEK293T cells were transfected with the indicated FLAG-Glis3 construct and Myc-Itch or empty vector. After 48 h, the cells were harvested and proteins examined by Western blot analysis using an anti-M2 FLAG-HRP or anti-GAPDH antibody. Bands were quantified as described in Materials and Methods. The average intensity of the bands of the Itch plus samples (n = 3) were normalized against that of GAPDH and plotted relative to the average intensity of the bands of the Itch minus samples. Representative images are shown below the histogram. B. HEK293T cells were transfected with FLAG Glis3 or the indicated zinc finger mutant and Myc-Itch or empty vector. After 48 h, cells were harvested and proteins examined by Western blot analysis with anti-M2 FLAG-HRP, anti-Myc, and goat anti-mouse-HRP antibodies. C. HEK293T cells were transfected with FLAG-Glis3 or the indicated ZF mutant along with p-mIP-696-Luc luciferase reporter. After 48 h cells were harvested and assayed for luciferase and ß-galactosidase activity and the normalized relative luciferase values (nRLU) were plotted. Each bar represents the mean +/- SEM. * indicates statistically different value from corresponding Myc empty vector control p < 0.02. # indicates statistically different value from corresponding WT Glis3 control p < 0.02. D. HEK293T cells were transfected with FLAG-Glis3 or the indicated zinc finger mutant along with CMV-HA-Ubiquitin and Myc Itch or empty vector. Cells were treated with 10 μM MG132 for 7 h prior to harvest. Co-immunoprecipitation was performed using an anti-M2 FLAG antibody and immunoprecipitated proteins examined by Western blot analysis using anti-M2 FLAG-HRP, anti-HA, and anti-Myc, and goat anti-mouse-HRP antibodies. E. HEK293T cells were transfected with FLAG Glis3 or the indicated zinc finger mutant and Myc Itch or empty vector. Cells were fixed in 4% paraformaldehyde, permeabilized, and stained with anti-M2 FLAG antibody followed by staining with anti-mouse Alexa 488. Protein localization was examined by fluorescence microscopy.

Since Itch interacts with and ubiquitinates Glis3, it was of interest to determine which regions were necessary for Itch-directed degradation of Glis3. Mutation of the *PY* ^*461*^ motif stabilized Glis3 in the presence of Itch as did deletion of the N-terminus (ΔN496), which includes the *PY* ^*461*^ motif (Fig 7A). In contrast, deletion of the C-terminus beyond the ZFD (ΔC653) had a negligible effect on Itch-mediated degradation of Glis3. These observations are consistent with our data showing that interaction of Itch with the Glis3 N-terminus, but not its C-terminus, which includes the transactivation domain of Glis3, is required for the Itch-mediated degradation of Glis3. Interestingly, even though the *PY* ^*461*^ motif is central to Glis3 degradation by Itch, the Glis3-ΔC480 mutant, which lacks the ZFD, was stable in the presence of Itch despite its interaction with and ubiquitination by Itch. These findings indicated that in addition to the *PY* ^*461*^ motif, the ZFD played a role in Itch-mediated destruction. To further analyze the requirement of the ZFD in the Itch dependent degradation of Glis3, the effect of disruption of the tetrahedral configuration of each zinc finger on Glis3 stability was examined. As seen in Fig 7B, mutation of either ZF3 or ZF4, but not ZF1, ZF2, or ZF5 inhibited the degradation of Glis3 by Itch. Since DNA binding and transactivation was ablated in all five zinc finger mutants ([33] and Fig 7C), loss of these two functions could not account for the requirement of ZF3 and ZF4 in Itch-mediated degradation. Additionally, ZF3 and ZF4 mutants were capable of interacting with Itch (data not shown) and were polyubiquitinated by Itch (Fig 7D) suggesting that the ZF mutations did not change the ubiquitination of Glis3 by Itch, but instead might affect the recognition of Itch-modified Glis3 by the proteasome. The Glis3 ZFD has been shown to be required for nuclear localization [33]; however, Glis3 nuclear localization was not affected by the ZF3 or ZF4 mutations (Fig 7E) suggesting that the observed stability of the mutants in the presence of Itch is not due to exclusion from the nucleus. Collectively, these data suggest that the ZFD of Glis3 is required for Itch-mediated degradation of the protein in addition to the N-terminal *PY* ^*461*^ motif in a manner that is not dependent on Glis3 nuclear localization or DNA binding.

### Itch inhibits Glis3-mediated transcription through degradation of Glis3 protein

**Fig 8:**
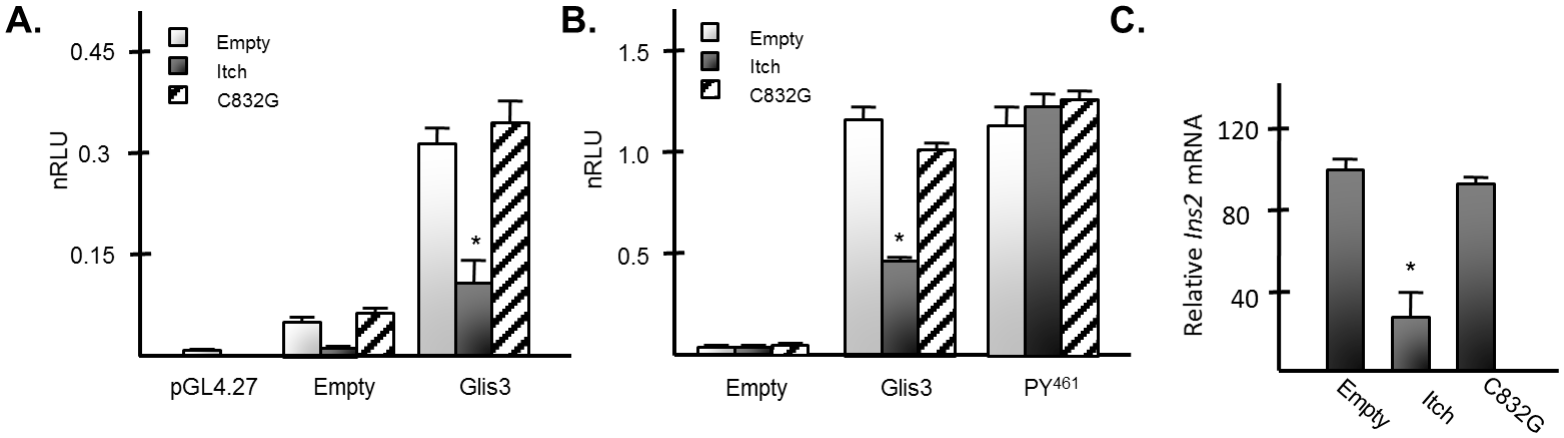
Itch inhibits Glis3-mediated transactivation of target genes. A. INS1 832/13 cells were transfected with pGL4.27 or p3xGlisBS-Luc, FLAG-Glis3, the *PY* ^*461*^ mutant, or empty vector, and Myc-Itch, the C832G mutant, or empty vector as indicated. After 48 h, cells were assayed for luciferase and β-galactosidase activity and the normalized relative luciferase activity (nRLU) was calculated and plotted. Each bar represents the mean +/- SEM. * Indicates statistically different value from corresponding Myc empty vector control p < 0.02. B. HEK293T cells were transfected with p-mIP-696-Luc, FLAG-Glis3, the *PY* ^*461*^ mutant, or empty vector, and Myc-Itch, the C832G mutant, or empty vector as indicated. After 48 h cells were assayed as described for A. Each bar represents the mean +/- SEM. * Indicates statistically different value from corresponding Myc empty vector control p < 0.02. C. INS1 832/13 cells were transfected with Myc-Itch, the C832G mutant, or empty vector as indicated. After 48 h, RNA was collected and rIns2 mRNA was measured by qRT-PCR analysis. Each bar represents relative *Ins2* mRNA normalized to 18s rRNA +/- SEM. * Indicates statistically different value compared to empty vector control p < 0.02.

Because Glis3 functions as a transcriptional activator for several target genes, including insulin, we were interested in determining whether Itch had any effect on Glis3-mediated transcriptional activation. Itch, but not the catalytically inactive mutant inhibited the transactivation of a Luc reporter driven by 3 tandem copies of the GlisBS in INS1 832/13 pancreatic β cells (Fig 8A). Importantly, Itch expression reduced activation of the reporter both in the presence or absence of exogenous Glis3 suggesting that it likely promotes the degradation of endogenous Glis3 in INS1 832/13 cells. Consistent with these results, luciferase reporter assays performed in HEK293T cells using the mouse *Ins2* promoter driven reporter construct, p-mIP-696-Luc, demonstrated that exogenous Itch expression again significantly reduced Glis3-mediated reporter activation (Fig 8B). Activation of the *Ins2* reporter by Glis3 was not inhibited by co-expression of the catalytically inactive Itch C832G mutant. Moreover, Itch did not inhibit the transactivation capacity of the Glis3 *PY* ^*461*^ mutant, which was stable in the presence of exogenous Itch expression. Together, these data indicate that the ligase activity of Itch as well as its interaction with the *PY* motif of Glis3 is required for the inhibition of Glis3 transcriptional activation and that the decreased level of activation is at least in part due to reduced protein levels following Itch-mediated degradation of Glis3. It further indicates that the effect of Itch is specific for Glis3 and not due to an effect on the general transcriptional machinery. In order to determine whether Itch had a similar effect on endogenous insulin transcription in INS1 cells, Myc-Itch or Myc-Itch C832G was transiently transfected into INS1 832/13 cells and their effect on r*Ins2* mRNA expression examined by QRT-PCR. Indeed, expression of Itch resulted in a > 60% decrease in *Ins2* mRNA, whereas Itch C832G expression had little effect (Fig 8C). In contrast, Smurf2 or NEDD4 expression did not significantly alter the activation of r*Ins2* transcription by Glis3 (S4A and S4B Fig).

## Discussion

Glis3-mediated transcriptional activation is likely regulated at multiple levels, including transcriptional, translational and post-translational mechanisms that control Glis3 protein activity and/or its expression level. We previously reported that the central region of Glis3 containing the ZFD and the C-terminal transactivation domain are essential for Glis3-mediated transcriptional activation [1,33]. In this study, we discover a new function for the N-terminus of Glis3 in the regulation of Glis3 stability and its transcriptional activity. Using the N-terminus of Glis3 as bait, we identified by GeLC-MS and Y2H analyses a number of novel Glis3 interacting partners, including several members of the Nedd4/Rsp5 family of HECT E3 ubiquitin ligases. We demonstrate that the HECT E3 ubiquitin ligase, Itch, promotes the polyubiquitination of Glis3, thereby targeting Glis3 for increased proteolytic degradation by the proteasome. Consequently, this leads to a substantially reduced Glis3 transcriptional activity as indicated by the decrease in Glis3-mediated activation of the m*Ins2* promoter as well as the transactivation of a reporter driven by 3xGlisBS. Itch and other E3 ligases have been reported to regulate the activity of various transcription factors and biological processes through different mechanisms [34]. For example, increased ubiquitination of p73 by Itch has been demonstrated to promote its degradation and consequently its transcriptional activity and function [35], while the hedgehog transcription factor Gli1 is targeted by Numb for Itch-dependent ubiquitination thereby inhibiting growth and promoting cell differentiation [36]. Itch has also been reported to interact with and target the pluripotency-associated transcription factor Oct4 for ubiquitination thereby affecting embryonic stem cell self renewal [37].

The inhibition of Glis3 activity by Itch was dependent on the interaction of Glis3 with and its ubiquitination by Itch since Itch had little effect on the transcriptional activity of the Glis3 mutant in which the Itch-interaction motif was mutated. Moreover, a catalytically inactive Itch mutant had little effect on Glis3-mediated transactivation. These observations are consistent with the conclusion that the reduction in transcriptional activity of Glis3 by Itch was likely related to decreased levels of Glis3 protein. Similarly, overexpression of Itch, but not the catalytically inactive mutant, in rat insulinoma INS1 832/13 cells resulted in a decrease in endogenous r*Ins2* mRNA expression presumably due to proteolytic degradation of endogenous Glis3. It is tempting to speculate that this regulation of Glis3 protein levels by Itch could serve as a mechanism for fine-tuning Glis3 function in pancreatic beta cells and might be part of the transcriptional control of insulin gene expression. In this context, it is interesting to note that heterozygous Glis3 null mice, which presumably express half the amount of Glis3, are more susceptible to developing glucose intolerance supporting the idea that reduced Glis3 protein levels can result in altered beta cell function and increased risk developing diabetes. Additionally, beta cell-specific KO of Glis3 in adult mice results in the development of hyperglycemia due to an almost total loss of insulin production [6]. The critical role for Glis3 in maintaining ß cell function is supported by GWAS studies implicating *GLIS3* as a risk locus for type 1 and type 2 diabetes [11–17]. It interesting to mention that the ubiquitin proteasome system plays an important role in the maintenance of pancreatic ß cell function and in islet dysfunction associated with type 2 diabetes [38]. It modulates the stability and activity of Pdx-1 and MafA, transcription factors with critical roles in regulating ß cell functions [39–40]. Studies of Itch knockout mice indicated a role for Itch in autoimmune disease and metabolic syndrome [41]. In addition to the pancreas, Itch, which is widely expressed, may additionally regulate Glis3 activity and function in several extrapancreatic tissues, such as kidney and osteoblasts. Both Glis3 and Itch have been implicated in, respectively, promoting or inhibiting osteoblast differentiation [2,42] and both proteins have been linked to the transcriptional mediator TAZ, which has been linked to the development of polycystic kidney disease as we reported for Glis3 [4,43–44]. Although these studies suggest possible links between Glis3, Itch and the physiological functions of Glis3, future studies are needed to further characterize the relationship between Itch-mediated degradation of Glis3 and the generation of pancreatic ß cells and the development Glis3-associated diseases, including type I and type 2 diabetes, osteopenia, and polycystic kidney disease.

Itch is not a constitutively active ligase since intramolecular interactions between its HECT and WW domains keeps Itch in an inactive conformation [36]. Itch can be activated through different mechanisms involving protein-protein interactions or post-translational modifications [36,45–46]. Interaction with Numb, a protein that plays an important role in development and lineage determination, releases Itch from its autoinhibitory conformation leading to Itch activation [36]. However, co-expression with Numb had little influence on Itch-mediated ubiquitination of Glis3 (not shown) suggesting that Itch might already be activated either by endogenous Numb or be activated by a different mechanism. It has been reported that Itch activity can also be controlled by different kinase signaling pathways [47]. The interaction of Glis3 with Itch was greatly dependent on a PPxY motif consistent with the consensus WW-domain interaction motifs previously described [34]. Mutation of the *PY* ^*461*^ motif did not completely eliminate the interaction or Itch-mediated ubiquitination of the protein however, when both Itch and Glis3 were overexpressed. Nonetheless, no association between Itch and regions outside the N-terminus of Glis3 were observed and mutation of the *PY* ^*461*^ motif in the context of the N-terminus alone was sufficient to eliminate both interaction and ubiquitination. WW-domain containing proteins have been reported to interact with phospho-serine/phospho-threonine-proline motifs or proline-rich stretches containing glycine or arginine [30,48–50]. For example, Di Marcotullio, et al. have demonstrated that eliminating Itch interaction with Gli1 required mutating a combination of PPxY and pSP motifs in the C-terminus of Gli1 [36], while others have shown that Itch can interact with the SH3 domain of endophilin A1 through a proline-rich region in its N-terminus [51]. Importantly, the interaction between Glis3 with endogenous Itch seemed to be almost completely ablated by the *PY* ^*461*^ mutation (Fig 2F) indicating that the interaction between Glis3 and Itch largely depends on a short region containing the *PPPY* ^*461*^ motif. WW-domains have been reported to make contacts with proline residues flanking the core PPxY motif of substrates [52–54]. The *PY* ^*461*^ motif is flanked on either side by a number of conserved proline residues interspersed with non-polar or basic residues. It is possible that Itch may make weak contacts with these flanking regions thereby enhancing its affinity for the *PY* ^*461*^ motif (S1A Fig). The weak interactions with *PY* ^*461*^ flanking regions may play a more prominent role at high Itch concentrations as under conditions of exogenous Itch overexpression.

Disruption of the core *PY* ^*461*^ motif of Glis3 by either mutation or removal of the motif by truncation was sufficient to stabilize Glis3 in the presence of the ubiquitin ligase. Interestingly, disruption of the tetrahedral configuration of Glis3 ZF3 and ZF4 protected Glis3 against Itch-directed degradation without significantly affecting the nuclear localization of Glis3, its interaction with Itch, or its Itch-mediated ubiquitination. These observations suggest that, in addition to requiring the *PY* ^*461*^ motif, Itch-mediated degradation also required the presence of the Glis3 ZFD. Although mutation of ZF3 and ZF4 disrupted DNA binding and transactivation by Glis3, so too did mutation of ZF1-2 and 5 without affecting protein degradation by Itch. These data indicate that targeted destruction of Glis3 by Itch does not appear to require DNA binding or transactivation by Glis3 but may require the presence of ancillary proteins that interact with the ZFD of Glis3. Indeed, previous reports have identified substrate-transferring proteins that may bind ubiquitinated substrates and facilitate the transfer of targeted proteins to the proteasome [55]. This hypothesis is supported by the fact that ZF3-4 mutants of Glis3 have higher levels of basal ubiquitination in the absence of Itch, suggesting that ubiquitinated Glis3 may not be turning over (Fig 7D). Alternatively, transfer of ubiquitin to a specific Lys residue within the zinc finger domains of Glis3 may be required for proper recognition by the proteasome. Although Itch does appear capable of transferring ubiquitin to the N-terminus of Glis3 lacking the ZFD, previous reports have indicated that ubiquitin can be promiscuously transferred to even non-native Lys residues by an E3 ubiquitin ligase [56]. Maximally efficient degradation of Glis3 by Itch may therefore require interaction with a complex of proteins associating with the Glis3 ZFD in addition to the N-terminal *PY* ^*461*^ motif.

It is well established that substrates are directed to the 26S proteasome through polyubiquitination, which occurs when chains of ubiquitin attached to the substrate by connecting subsequent ubiquitin molecules to one of seven lysine residues within the preceding ubiquitin molecule [21]. Most often proteins are targeted to the 26S proteasome for degradation when modified with K48-linked polyubiquitin chains or less commonly, K29-linked chains [57]. Surprisingly, despite evidence indicating that Itch targets Glis3 for proteasomal degradation, we found that mutation of ubiquitin K63, but not K48 significantly reduced the amount of polyubiquitinated Glis3 in the presence of Itch. Previous reports have indicated that Itch, like its yeast homolog, *Rsp5*, preferentially utilizes K63 linkage, while other studies have demonstrated that K63-linked polyubiquitin chains can interact with the 26S proteasome and target proteins for degradation [58–62]. Importantly, use of the ubiquitin K63 mutant did not totally eliminate Glis3 polyubiquitination by Itch, indicating that other lysine residues may be able to substitute for K63. Others have reported Itch-mediated degradation of substrate proteins by K29-linked ubiqutination [57], which was not tested in this study. Further, mixed-chain linkages have been reported [63] and it is possible that Glis3 ubiquitination involves a more complex mechanism involving mixed or branched chain ubiquitin moieties.

In addition to Itch, several other WW-domain containing HECT E3 ubiquitin ligases, including Nedd4, Smurf1-2, and Wwp2, were identified by Y2H analysis as potential interacting partners of Glis3 through their WW domains. The association between Smurf2 and NEDD4 with Glis3 seemed to be similarly through the *PY* ^*461*^ motif. The interactions between Smurf2 and NEDD4 with Glis3 were considerably weaker than observed for Itch, while NEDD4 failed to interact with full-length Glis3 altogether. It is of interest to note that Smurf2 and Itch contain non-canonical WW-domains (containing a tyrosine or phenylalanine substitution for a conserved tryptophan) that involve their binding pocket tryptophans responsible for recognizing polyproline motifs [28], while NEDD4 does not contain any non-canonical WW-domains. Thus, these differences in WW-domain structure might result in different affinities for the core *PPxY* motif in Glis3. In contrast to Itch, neither Smurf2 nor NEDD4 increased Glis3 polyubiquitination nor caused a change in the total level of Glis3 protein. It is likely that lack of polyubiquitination and subsequent degradation of Glis3 by Smurf2 and NEDD4 might be due to a lower affinity for Glis3. In addition, differences in subcellular localization of HECT ubiquitin ligases might play a role in how effective they bind Glis3. Itch, which like Glis3, can localize to the nucleus, might be able to bind Glis3 more effectively than Nedd4, which is largely membrane bound or cytosolic [64]. These characteristics together may reduce the efficiency with which these proteins ubiquitinate Glis3, such that only target a very small fraction of Glis3 is targeted for degradation by the proteasome without significantly changing the total level of Glis3 protein. Moreover, ubiquitination by different E3 ligases might affect Glis3 in distinct ways as has been reported for other proteins [35,65,66] and might relate to differences in the location or type of polyubiquitination of Glis3 as well as cell type. The functional consequence of polyubiquitination has been shown to also depend on the lysine residue within ubiquitin that is used for chain elongation [64–66]. For example, ubiquitination of the transcriptional factor p73 by Itch leads to increased degradation of p73, whereas NEDD-mediated ubiquitination results in p73 stabilization [35]. Further study is needed to determine the physiological relevance of the interactions between NEDD4 and Smurf2 with Glis3. It is interesting to note that in mice Smurf1/2 have been implicated in the regulation of planar cell polarity and renal fibrosis [67]. The latter might be relevant to the function of Glis3 in kidneys since the loss of Glis3 function leads to the development of polycystic kidney disease in both mice and humans [4,8]. Defects in planar cell polarity and at later stages fibrosis are prominent features of this disease.

In conclusion, in this report we demonstrate for the first time that Glis3 interacts with several members of the HECT E3 ubiquitin ligase family. Interaction with Itch leads to increased polyubiquitination and proteasomal degradation of Glis3, which consequently results in reduced Glis3 transcriptional activation, including that of the insulin promoter. Our study identifies Itch as a novel negative regulator of Glis3-mediated transcription and Glis3 functions. Glis3 plays a critical role in beta cell generation and insulin regulation and is implicated in the development of type 1 and 2 diabetes [3,5,18]. These findings, together with reports showing that ubiquitination of two other critical ß cell transcription factors, Pdx-1 and MafA [39,40], support the important role of ubiquitination in the control of ß cell functions.

## Supporting Information

S1 Fig HECT E3 ubiquitin ligases interact with Glis3 through a conserved PPxY motif. A. Alignment of the region surrounding the Glis3 PPxY motif from selected species. m = mus musculus; r = rattus norvegicus; h = homo sapiens; g = Gallus gallus; x = Xenopus tropicalis; d = Danio rerio; o = Oryzias latipes. Core PPxY motif is underlined. B. HEK293T cells were transfected with FLAG Glis3 or the indicated PPxY mutant and Myc empty vector or Myc-Itch-C832G. Co-immunoprecipitation was performed using a mouse anti-Myc antibody and immunoprecipitated proteins were examines by Western blot analysis using anti-M2 FLAG-HRP or anti-Myc and goat anti-mouse-HRP antibodies. C-D. HEK293T cells were transfected with FLAG-Glis3 or the PY461 mutant and the WW domains of Smurf2 or NEDD4 as indicated. Co-IP was performed as described in B.
(TIF)

S2 Fig Itch promotes polyubiquitination of Glis3. A-B. HEK293T cells were transfected with CMV-HA-Ubiquitin, FLAG-Glis3 or FLAG-Glis3-ΔC480 or their respective *PY* ^*461*^ mutants, and Myc-Itch or empty vector as indicated. Cells were treated with 10 μM MG132 for 6 h prior to harvest. Co-immunoprecipitation was performed using an anti-HA antibody and immunoprecipitated proteins were analysed by Western blot using a high affinity rat anti-HA antibody anti-M2 FLAG-HRP antibody goat anti-rat-HRP antibodies.
(TIF)

S3 Fig Itch induces K63-linked rather than K48-linked polyubiquitination of Glis3. HEK293T cells were transfected with FLAG-Glis3, Myc Itch, and HA-Ubiquitin or the K48R or K63R ubiquitin mutants as indicated. Cells were treated with 10 μM MG132 for 6 h prior to harvest. Co-immunoprecipitation was performed using an anti-M2 FLAG antibody and immunoprecipitated proteins were analysed by Western blot using a high affinity anti-HA, anti-M2 FLAG-HRP, anti-Myc, and goat anti-mouse-HRP antibodies.
(TIF)

S4 Fig Smurf2 or NEDD4 do not influence *Ins2* transcription in INS1 832/13 cells. A-B. INS1 832/13 cells were transfected with Myc-Smurf2 or Myc-NEDD4 or their respective catalytically inactive mutants as indicated. After 48 h, RNA was collected and r*Ins2* mRNA was measured by qRT-PCR analysis. Each bar represents relative *Ins2* mRNA normalized to 18s rRNA +/- SEM.
(TIF)

S1 Table Table of primers used in site-directed *in vitro* mutagenesis. Primers are shown 5’ to 3’. Reverse complement primers are not shown. Mutated bases are underlined and in bold font.
(DOCX)

S2 Table Table of Glis3 interacting partners determined by mass spectrometry. Protein symbol and ascension number is given for each protein. MW = molecular weight in kD.
(DOCX)
