# Supplementary figures and images for: HECT E3 Ubiquitin Ligase Itch Functions as a Novel Negative Regulator of Gli-Similar 3 (Glis3) Transcriptional Activity
Source: PLoS One. 2015 Jul 6;10(7):e0131303. doi: 10.1371/journal.pone.0131303 (PMC4493090; doi:10.1371/journal.pone.0131303)

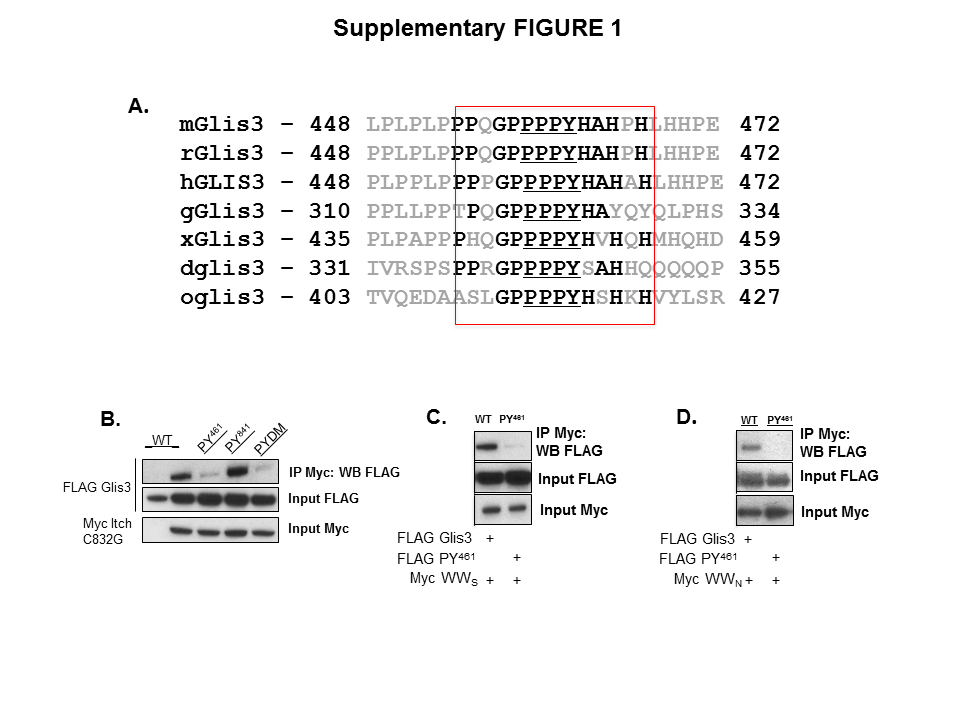

Supplement: S1 Fig — A. Alignment of the region surrounding the Glis3 PPxY motif from selected species. m = mus musculus; r = rattus norvegicus; h = homo sapiens; g = Gallus gallus; x = Xenopus tropicalis; d = Danio rerio; o = Oryzias latipes. Core PPxY motif is underlined. B. HEK293T cells were transfected with FLAG Glis3 or the indicated PPxY mutant and Myc empty vector or Myc-Itch-C832G. Co-immunoprecipitation was performed using a mouse anti-Myc antibody and immunoprecipitated proteins were examines by Western blot analysis using anti-M2 FLAG-HRP or anti-Myc and goat anti-mouse-HRP antibodies. C-D. HEK293T cells were transfected with FLAG-Glis3 or the PY461 mutant and the WW domains of Smurf2 or NEDD4 as indicated. Co-IP was performed as described in B. (TIF) [file pone.0131303.s001.tif]

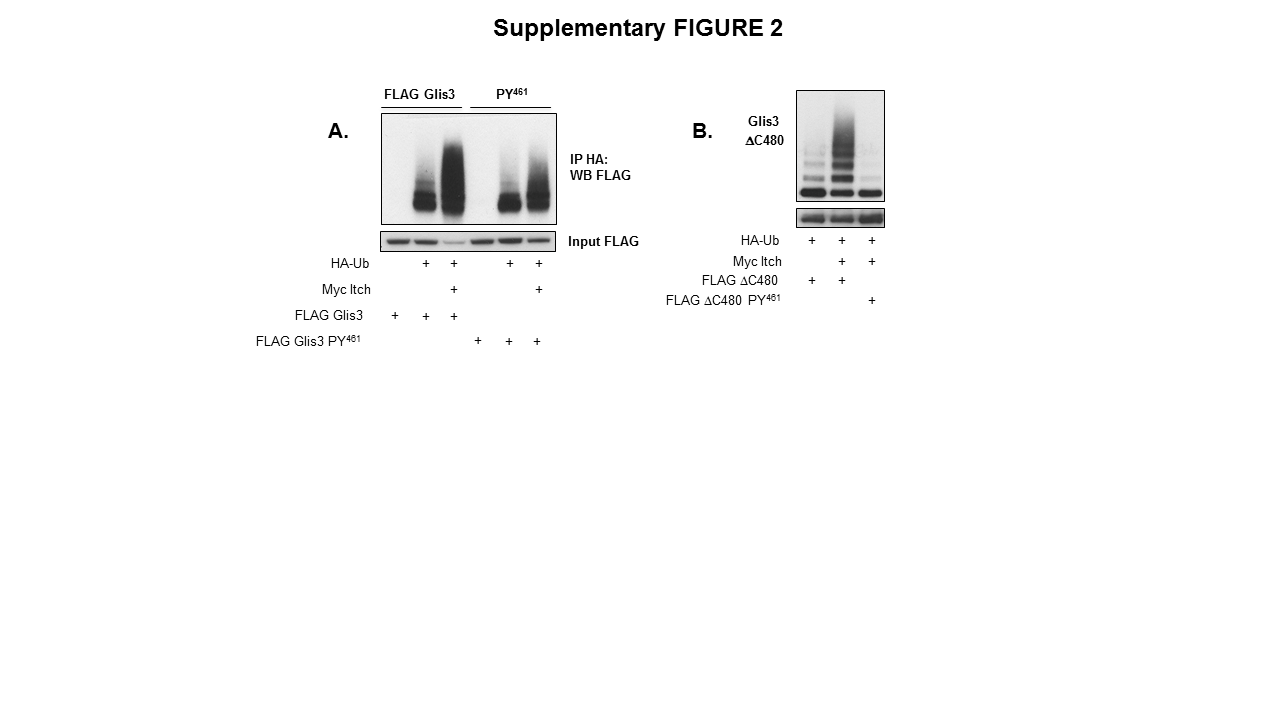

Supplement: S2 Fig — A-B. HEK293T cells were transfected with CMV-HA-Ubiquitin, FLAG-Glis3 or FLAG-Glis3-ΔC480 or their respective PY 461 mutants, and Myc-Itch or empty vector as indicated. Cells were treated with 10 μM MG132 for 6 h prior to harvest. Co-immunoprecipitation was performed using an anti-HA antibody and immunoprecipitated proteins were analysed by Western blot using a high affinity rat anti-HA antibody anti-M2 FLAG-HRP antibody goat anti-rat-HRP antibodies. (TIF) [file pone.0131303.s002.tif]

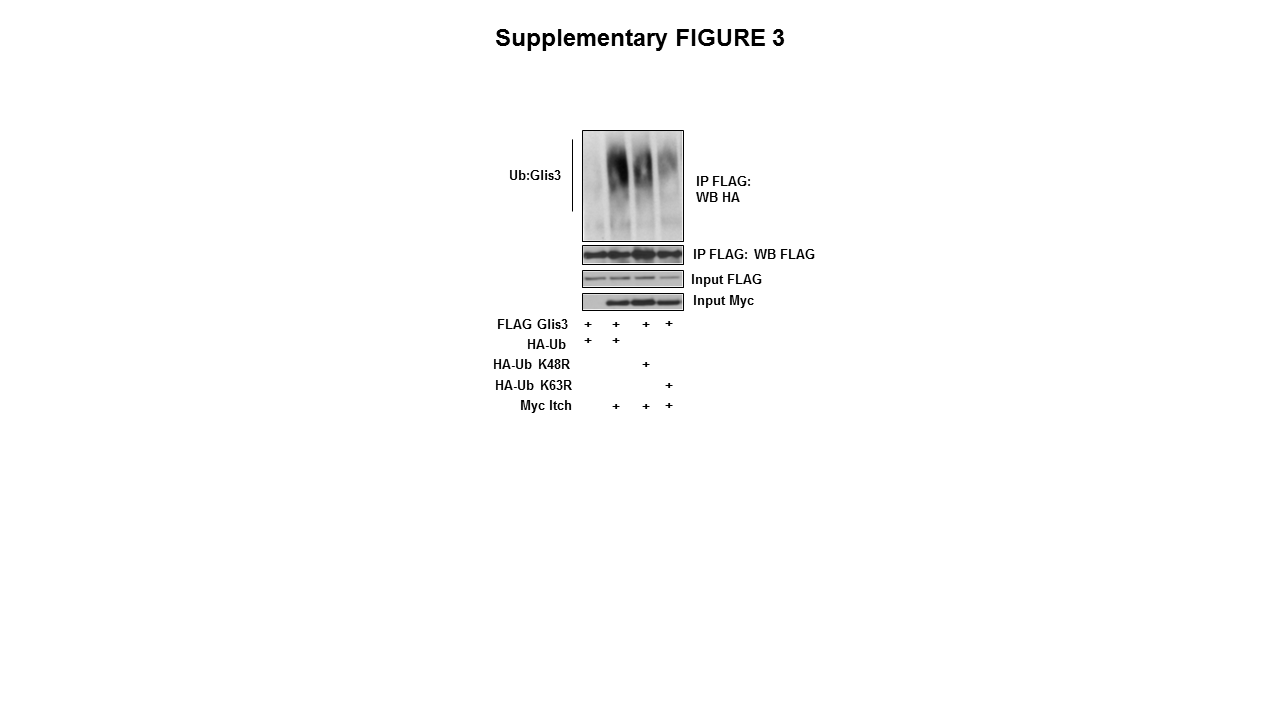

Supplement: S3 Fig — HEK293T cells were transfected with FLAG-Glis3, Myc Itch, and HA-Ubiquitin or the K48R or K63R ubiquitin mutants as indicated. Cells were treated with 10 μM MG132 for 6 h prior to harvest. Co-immunoprecipitation was performed using an anti-M2 FLAG antibody and immunoprecipitated proteins were analysed by Western blot using a high affinity anti-HA, anti-M2 FLAG-HRP, anti-Myc, and goat anti-mouse-HRP antibodies. (TIF) [file pone.0131303.s003.tif]

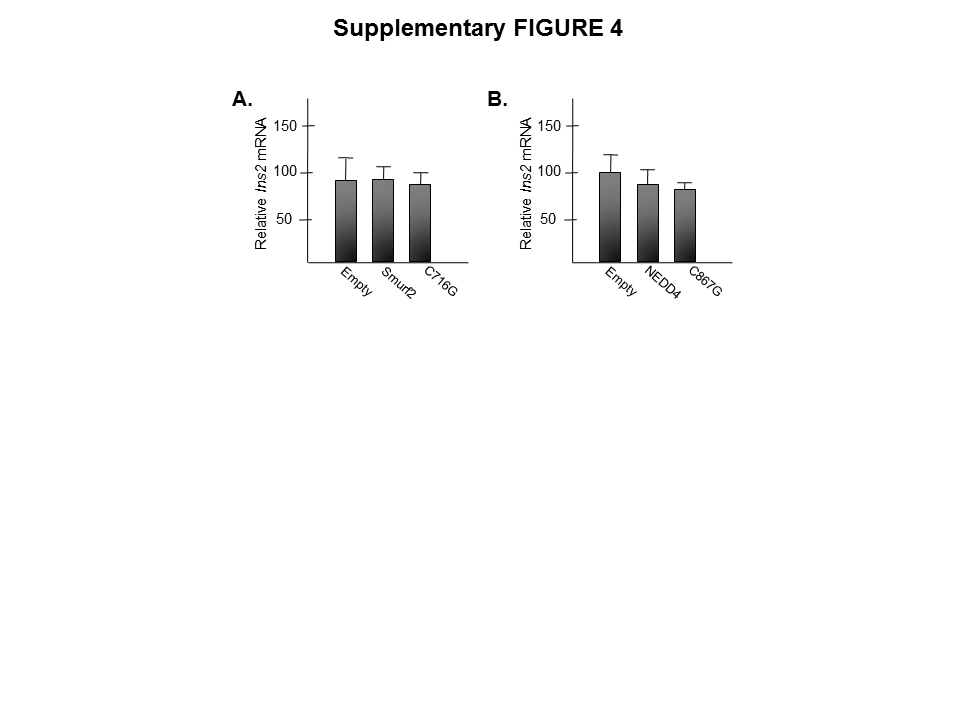

Supplement: S4 Fig — A-B. INS1 832/13 cells were transfected with Myc-Smurf2 or Myc-NEDD4 or their respective catalytically inactive mutants as indicated. After 48 h, RNA was collected and rIns2 mRNA was measured by qRT-PCR analysis. Each bar represents relative Ins2 mRNA normalized to 18s rRNA +/- SEM. (TIF) [file pone.0131303.s004.tif]
