# Supplementary material for: HECT E3 Ubiquitin Ligase Itch Functions as a Novel Negative Regulator of Gli-Similar 3 (Glis3) Transcriptional Activity
Source: PLoS One. 2015 Jul 6;10(7):e0131303. doi: 10.1371/journal.pone.0131303 (PMC4493090; doi:10.1371/journal.pone.0131303)
Supplement: S1 Table — Primers are shown 5’ to 3’. Reverse complement primers are not shown. Mutated bases are underlined and in bold font. (DOCX) [file pone.0131303.s005.docx]

**S1 Table.** **Table of primers used in site-directed *in vitro* mutagenesis.**

| **Primer Name** | **Primer Sequence** |
| --- | --- |
| Glis3 PY^461^ mut | 5’ – GGGTCCCCCA**G**CCCCA**GC**CCATGCCCATCC |
| Glis3 ZF1 mut | 5’ – GGGAAGCACTGC**GC**CCGTTGGATAGACTGC |
| Glis3 ZF2 mut | 5’ – GGAAGACTTCACG**GC**CTTCTGGACTGGC |
| Glis3 ZF3 mut | 5’ – GGAGAAGCCCAACAAG**GC**TACGTTCGAAGGCTGC |
| Glis3 ZF4 mut | 5’ – GGTGAGAAGCCATACTTG**GC**CCAGCATCCGGGCTGC |
| Glis3 ZF5 mut | 5’ - CTAAACCTTATGCT**GC**TCAAATTCCAGG |
| Ubiquitin K11R | 5’ – CCCTTACCGGCA**G**GACCATCACCC |
| Ubiquitin K29R | 5’ – CGAAAATGTGAAGGCCA**G**GATCCAGGATAAGG |
| Ubiquitin K48R | 5’ – GCTCATCTTTGCAGGCA**G**GCAGCTGGAAGATGG |
| Ubiquitin K63R | 5’ - GACTACAACATCCAGA**G**GGAGTCGACCCTGCACCTGGTCC |
| Smurf2 C716G | 5’ – CCGAAAGCCCACACT**G**GCTTCAATCGAATAG |
| Itch C832G | 5’ – CCAGAAGCCATACT**G**GTTTTAACCGCCTGGACC |
| NEDD4 C854G | 5’ – CCAAGAGCACACACC**G**GCTTCAATCGCCTGG |
