# Supplementary material for: HECT E3 Ubiquitin Ligase Itch Functions as a Novel Negative Regulator of Gli-Similar 3 (Glis3) Transcriptional Activity
Source: PLoS One. 2015 Jul 6;10(7):e0131303. doi: 10.1371/journal.pone.0131303 (PMC4493090; doi:10.1371/journal.pone.0131303)
Supplement: S2 Table — Protein symbol and ascension number is given for each protein. MW = molecular weight in kD. (DOCX) [file pone.0131303.s006.docx]

**S2 Table.** **Table of Glis3 interacting partners determined by mass spectrometry.**

| **Protein Name** | **MW** | **Ascension Number** |
| --- | --- | --- |
| TUBB | 49.7 | IPI00011654 |
| TUBA1C | 49.9 | IPI00218343 |
| MYH9 | 226.5 | IPI00019502 |
| MYH10 | 233.6 | IPI00391300 |
| CAD | 24.3 | IPI00301263 |
| SUFU | 54.1 | IPI00124718 |
| IRS4 | 133.8 | IPI00020729 |
| HSP90B | 83.3 | IPI00229080 |
| HSPA4 | 94.3 | IPI00002966 |
| HSPA4L | 94.5 | IPI00828021 |
| MSH6 | 152.8 | IPI00384456 |
| EEF1A1 | 50.2 | IPI00472724 |
| EEF2 | 95.3 | IPI00186290 |
| SERPINH1 | 46.4 | IPI00032140 |
| RUVBL1 | 50.3 | IPI00021187 |
| DNAJA1 | 44.9 | IPI00012535 |
| RCN1 | 38.9 | IPI00015842 |
| RCN2 | 39.1 | IPI00790214 |
| MCM7 | 81.3 | IPI00299904 |
| BAG2 | 23.8 | IPI00000643 |
| BAG5 | 56.0 | IPI00556027 |
| STUB1 | 34.9 | IPI00471361 |
| HIST1H2AA | 18.6 | IPI00559107 |
| DIS3 | 109.0 | IPI00746351 |
| TRIP13 | 48.6 | IPI00003505 |
| PFKP6 | 85.7 | IPI00231954 |
| PFKL6 | 90.6 | IPI00925520 |
| ITCH | 102.8 | IPI00061780 |
| GALK1 | 45.4 | IPI00019383 |
| EIF4A1 | 46.2 | IPI00025491 |
| MAD2L1 | 23.5 | IPI00012369 |
| PKM2 | 57.9 | IPI00479186 |
| PRMT5 | 72.7 | IPI00441473 |
| LEPRE1 | 90.6 | IPI00045839 |
| PANK4 | 86.0 | IPI00939845 |
